# Human papilloma virus: global research architecture assessed by density-equalizing mapping

**DOI:** 10.18632/oncotarget.25136

**Published:** 2018-04-24

**Authors:** Dörthe Brüggmann, Luise Kayser, Jenny Jaque, Matthias Bundschuh, Doris Klingelhöfer, David A. Groneberg

**Affiliations:** ^1^ Department of Obstetrics and Gynecology, Keck School of Medicine of USC, Los Angeles, CA 90033, USA; ^2^ Department of Female Health and Preventive Medicine, Institute of Occupational Medicine, Social Medicine and Environmental Medicine, Goethe-University, Frankfurt 60590, Germany

**Keywords:** HPV, human papilloma virus, publication, bibliometry, scientometry

## Abstract

Human papilloma virus (HPV) infection is linked to cervical cancer, which represents the world's fourth most common cancer in women. So far, no detailed map of the worldwide HPV research architecture has been constructed. Hence, this study focuses on the chronological development and geographical distribution of the global HPV-specific publications and evaluates citation-based parameters as well as socioeconomic features of the publishing countries.

In total, 29,330 HPV-related publications were identified. The US was the leading country with 12,270 publications. Only high-income-countries were found in the ranking of the fifteen most active countries with Germany, France, and Japan among the top five. Analysis of HPV research activity in relation to the economic strength demonstrated a lead position of Finland and Sweden with an average of 2248.78 and 1924.67 HPV-related publications per GDP in 1000 bn US-$, respectively. The most active upper-middle-income country was Mexico (416.78 HPV-related publications per GDP in 1000 bn US-$). India as lower-middle-income country reached a value of 279.78 HPV-related publications per GDP in 1000 bn US-$. Collaboration analysis pointed to the US as the center of the 4517 international HPV collaborations.

The worldwide HPV-research landscape is dominated by North American and Western European countries. By contrast, a high prevalence of HPV-related cervical cancer is documented for low-income countries. Hence, HPV-related public health interventions and prevention research specifically tailored to these countries needs to be fostered by monetary support and international collaborations.

## BACKGROUND

The Human papillomavirus (HPV) plays a major role in various fields of oncology [1–11]. It is a resistant, ubiquitous virus that survives in the environment without a host. A chronic infection with high-risk genotypes of HPV causes around 70% of all squamous cervical cancers. Also, the pathogenesis of anogenital and oropharyngeal cancers has been linked to HPV [12]. From a global viewpoint, HPV is responsible for about 4.5% (630,000) of all new cancer cases worldwide causing a problem predominately in developing countries [13]. But also in industrialized nations, HPV infections and associated malignancies constitute a relevant public health burden. In the United States of America (US), nearly 80 million people are estimated to have a prevalent HPV infection [14]. The American Cancer Society predicts for 2018 that 13,240 women will receive a diagnosis of cervical cancer; 4170 deaths will be linked to this malignancy (American Cancer Society Facts and Figures 2015).

Since the identification of HPV and its characterization as oncogenic agent, the related field of biomedical research grew rapidly. Besides screening practices and possible treatments, scientists are focusing upon public health issues concerning the prevention of HPV-associated diseases. Particularly, vaccine research merits a high academic and commercial interest, which has created an additional booster for HPV-related research. Concerning vaccination strategies, two vaccines are commonly used around the world, a bivalent vaccine targeting HPV 16 and 18 genotypes and a quadrivalent vaccine against HPV types 6, 11, 16, and 18 [12, 15, 16]. Clinical trials demonstrated that three vaccine doses provide up to 100 % protection against a cervical infection with HPV and related precancerous lesions in women aged 15-26 years with no prior exposure to the virus [12].

The global impact of HPV infections and their multi-layered clinical consequences have translated into an immediate call for action in the fields of public and global health. In this respect, the introduction of HPV vaccines was first only feasible in selected high-income countries due to high expenditures of vaccination costs [12]. Since prices dropped from 2011 onwards, some middle-income countries implemented HPV vaccination programs in their health systems [12]. In 2012, finally, the GAVI Alliance realized funding of pilot HPV vaccine projects in low-income countries [12, 17, 18]. This initiative will hopefully translate into widespread policy changes in underserved countries. Here, primary prevention is the key to effectively lower the population burden related to cervical cancer death because access to screening and treatment is extremely limited for local women.

Despite the enormous significance of HPV, its related conditions and their prevention, no systematic evaluation has been done yet to detail the global architecture of the worldwide scientific output associated with HPV. Efforts are lacking to understand developments in the related literature that can be utilized to plan future research activities or funding strategies according to identified shortcomings. Therefore, the NewQIS (“New Quality and Quantity Indices in Science”) project platform [19] decided to analyze this respective field of medicine. As primary aim of this study, a picture of the global HPV research architecture was created, with regard to overall HPV research activity, its geographical and chronological trends, aspects of research quality and the economic background of the research.

## RESULTS

### General parameters

**Figure 1:**
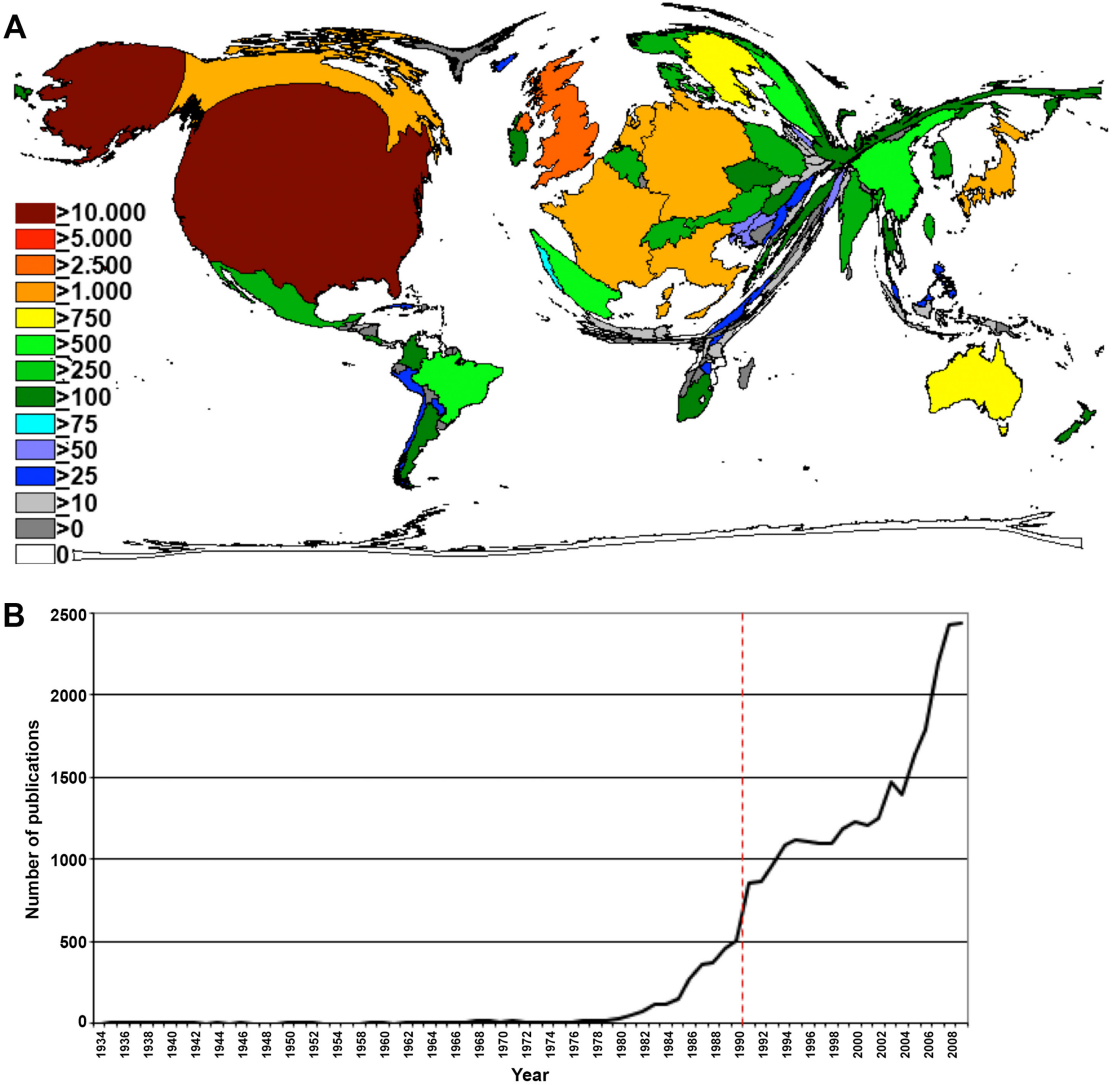
Research output **(A)** Number of published items per year. **(B)** Density equalizing map of the global HPV research activity between 1900 and 2009. Colors and territorial sizes indicate numbers of HPV publications per country.

Between 1900 and 2009 a total of 29,330 publications (p) were identified. 96.3 % of the HPV-specific items were published in English, 1.5 % in German and 1.2 % in French. 28,966 out of all HPV articles were authored between 1980 and 2009. There was a continuous increase in publication activities throughout the 1980s; the number of publications in 1989 was 13 times higher (p=463) than in 1980. For 2009, we documented a total of 2439 HPV-specific publications (Figure 1A). Over time, the number of participating authors per item increased. While we found an average of 3.7 participating authors per HPV related paper in 1980, the average number of authors increased to nearly six in 2009.

### Country-specific analysis

In the country-specific analyses, the United States (US) represented the country with the highest HPV research activity. US-American authors published a total of 12,270 publications. The US was followed by the United Kingdom (UK, p=2644), Germany (p=2463), France (p=1670), Japan (p=1316), Italy (p=1292), Canada (p=1076), the Netherlands (p=1015) and Australia (p=789). DEMP analysis demonstrated a corresponding distortion of the world map with a focus on North America and Western Europe (Figure 1B).

**Figure 2:**
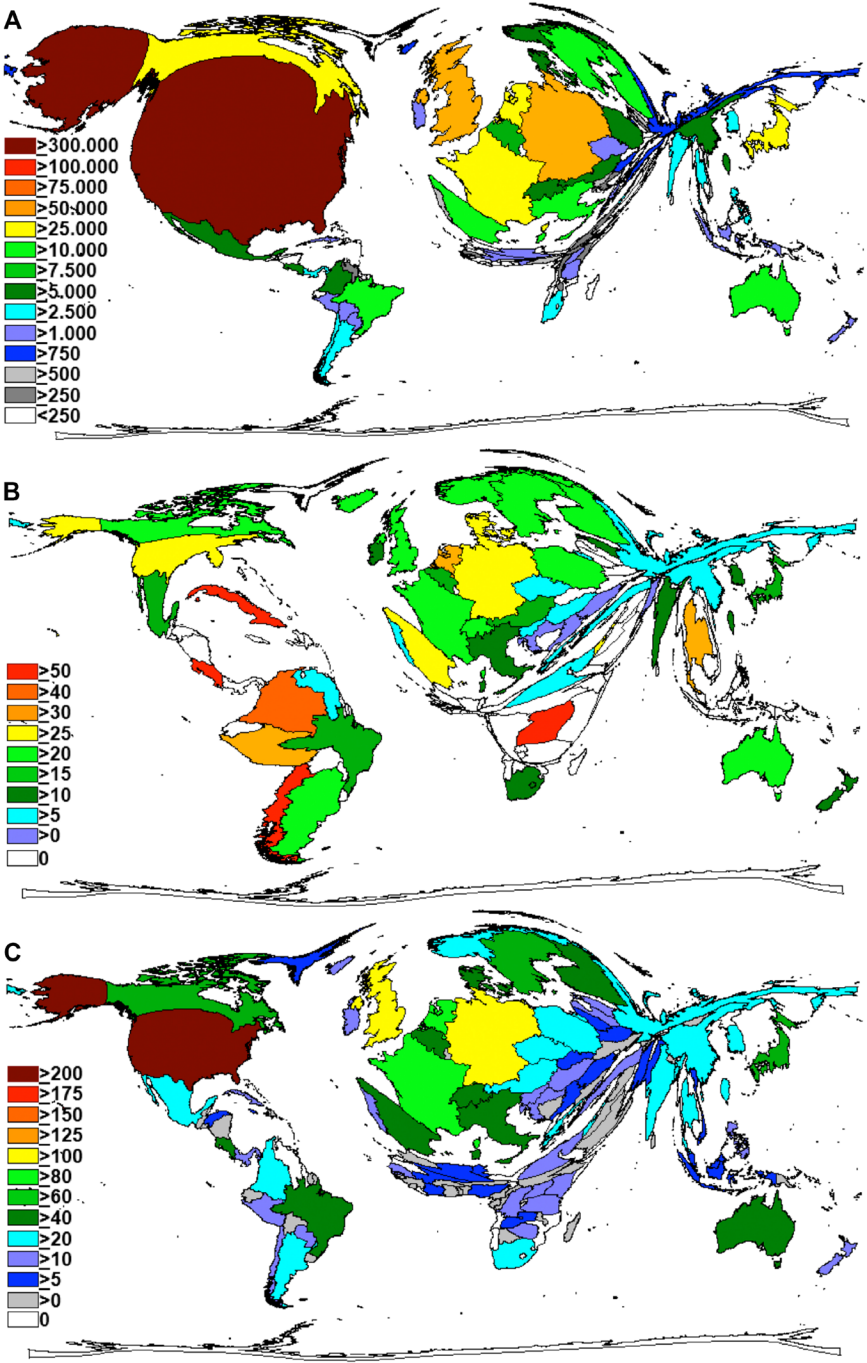
Density equalizing maps projections **(A)** Total number of HPV-specific citations per country. **(B)** Levels of HPV-specific citation rates of countries (threshold 30 publications). **(C)** Levels of HPV-specific h-indices of countries.

The total citation number analysis listed the US also on position 1 (Figure 2A). The nation was followed by the UK and Germany with over 50,000 citations of HPV-specific publications each as well as France, Canada and the Netherlands with more than 25,000 citations. The analysis of the HPV-specific h-index for countries (termed the modified h-index) revealed a similar picture. We showed a leading position of the US, followed by the UK and Germany (Figure 2B). By contrast, when the citation number per research item (citation rate) were analyzed and a threshold of at least 30 HPV-specific publications per country was applied, the citation rate ranking was led by Chile, Costa Rica and Cuba and Uganda with more than 50 citations per HPV-specific publications. Also, scientists from Columbia and Peru authored publications with citation rates of over 40 and 30 citations per HPV-specific publication, respectively. When the threshold was increased to i.e. at least 100 HPV-specific publications per country, these countries disappeared from the ranking (Figure 2C).

### Socio-economic analysis

When we related the gross domestic product as an indicator for total economic strength to the countries´ HPV research activities, all of the fifteen top ranked countries could be identified as high-income countries according to the World Bank categories (Table 1). In this ranking, the two Scandinavian countries Finland and Sweden were ranked first and second with an average of 2254.48 and 1817.73 HPV-related publications per GDP in 1000 bn US-$ (Socio-Economic HPV Index 1 = SEI_1_), respectively. They were followed by the Netherlands (SEI_1_=1183.08), and the UK (SEI_1_=1116.97). The US were ranked 7^th^ with a SEI_1_ of 850.98. In this calculation, the highest upper-middle-income country was Mexico with 416.78 HPV-related publications per GDP in 1000 bn US-$. India as lower-middle-income country reached a value of 288.53 and China as an upper-middle-income country a value of 87.71 HPV-related publications per GDP in 1000 bn US-$.

**Table 1:** Socio-economic analysis of HPV research of the ten most active countries

Country	Publ.	GDP in Tbn UDS	Publ./GDP in Tbn USD	Rank	GDP per Capita in TUSD	Publ./GDP per Capita in TUSD	Rank	Pop.in mill.	Publ./Pop.in mill.	Rank
Finland	567	0,25	2254,48	HI1	34,03	16,66	HI9	5,34	106,20	HI1
Sweden	781	0,43	1817,73	HI2	357,16	2,19	HI15	9,30	83,99	HI2
Netherlands	1015	0,86	1183,08	HI3	37,68	26,94	HI6	16,53	61,40	HI3
UK	2644	2,37	1116,97	HI4	24,78	106,72	HI2	62,28	42,46	HI5
Belgium	479	0,48	988,54	HI5	32,93	14,55	HI10	10,80	44,37	HI4
Australia	789	0,93	850,98	HI6	63,90	12,35	HI12	21,69	36,37	HI9
USA	12270	14,42	850,98	HI7	47,58	257,90	HI1	306,77	40,00	HI6
Austria	315	0,40	792,26	HI8	34,65	9,09	HI13	8,34	37,75	HI8
Canada	1076	1,37	784,74	HI9	47,95	22,44	HI8	33,63	32,00	HI10
Germany	2463	3,42	720,60	HI10	30,27	81,38	HI3	81,90	30,07	HI11
Taiwan^*^	474	0,71	664,14	HI11	34,10	13,90	HI11	22,97	20,63	HI14
France	1670	2,69	619,94	HI12	30,29	55,13	HI4	64,71	25,81	HI12
Italy	1292	2,19	591,26	HI13	26,70	48,39	HI5	59,10	21,86	HI13
Switzerland	301	0,54	557,89	HI14	76,03	3,96	HI14	7,74	38,87	HI7
South Korea	492	0,90	545,49	HI15	24095,94	0,02	HI17	49,31	9,98	HI17
Mexico	373	0,89	416,78	UMI1	101,13	3,69	UMI3	115,51	3,23	UMI1
Spain	569	1,50	379,57	HI16	23,31	24,41	HI7	46,36	12,27	HI15
Brazil	613	1,67	367,72	UMI2	8,21	74,63	UMI2	194,90	3,15	UMI2
India	382	1,32	288,53	LMI1	61,19	6,24	LMI1	1214,27	0,31	LMI1
Japan	1316	5,23	251,56	HMI17	3750,35	0,35	HI16	128,05	10,28	HI16
China^*^	701	7,99	87,71	UMI3	6,00	116,83	UMI1	1338,61	0,52	UMI3

^*^ aus World Factbook.

T= Thousand.

Publ. = Publications.

Pop. = Population.

Sources for GDP (Current Prices in 1000 bn US Dollars) and GDP per capita (Current Prices in 1000 US Dollars) in 2009: World Bank (China/Taiwan figures are taken from the World Factbook, Population numbers in 2009, GDP and GDP per Capita in 2008. HI, high- income country, UMI, upper-middle-income country, LMI, lower-middle-income country according to the World Bank categories. Threshold level of at least 300 HPV-related publications.

When the HPV-specific publications were related to the relative economic power indicator GDP per capita in 1000 US-$ (Socio-Economic HPV Index 2 = SEI_2_), the US were ranked at the top position with a value of SEI_2_=257.90, followed by China (SEI_2_=116.83), the UK with a value of SEI_2_=106.72, and Germany (SEI_2_=81.38).

Looking at the HPV-related publications per number of inhabitants (SEI_3_) the following order can be determined: Finland (SEI_3_=106.20), Sweden (SEI_3_=83.99), Nethrlands (SEI_3_=61.40), Belgium (SEI_3_=44.37), UK (SEI_3_=42.46) and the US at rank 6 (SEI_3_=40.00).

### HPV subject area analysis

We could identify a total of 57 different subject categories assigned to HPV research. The leading subject category was the category “Oncology” with 7098 HPV publications, which was not surprising. It was followed by “Pathology” with 3661 HPV-related publications. We found “Obstetrics and Gynecology” and “Virology” at positions three and four with 3395 and 3352 HPV-related publications, respectively. “Public health” was at position 12 with about 1100 HPV-specific publications.

**Figure 3:**
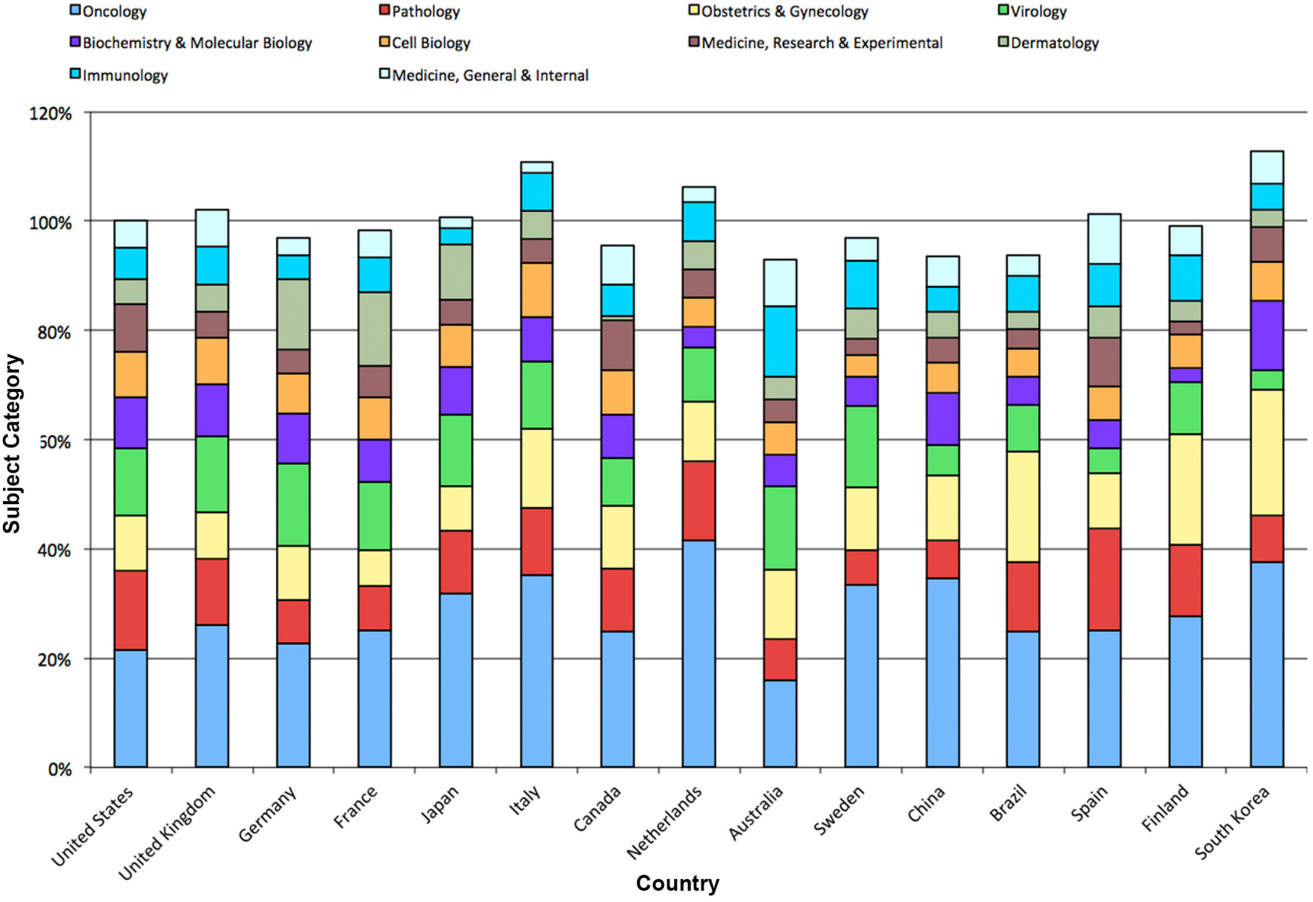
Subject area analysis of HPV research Relative proportions of subject areas in most active countries.

**Figure 4:**
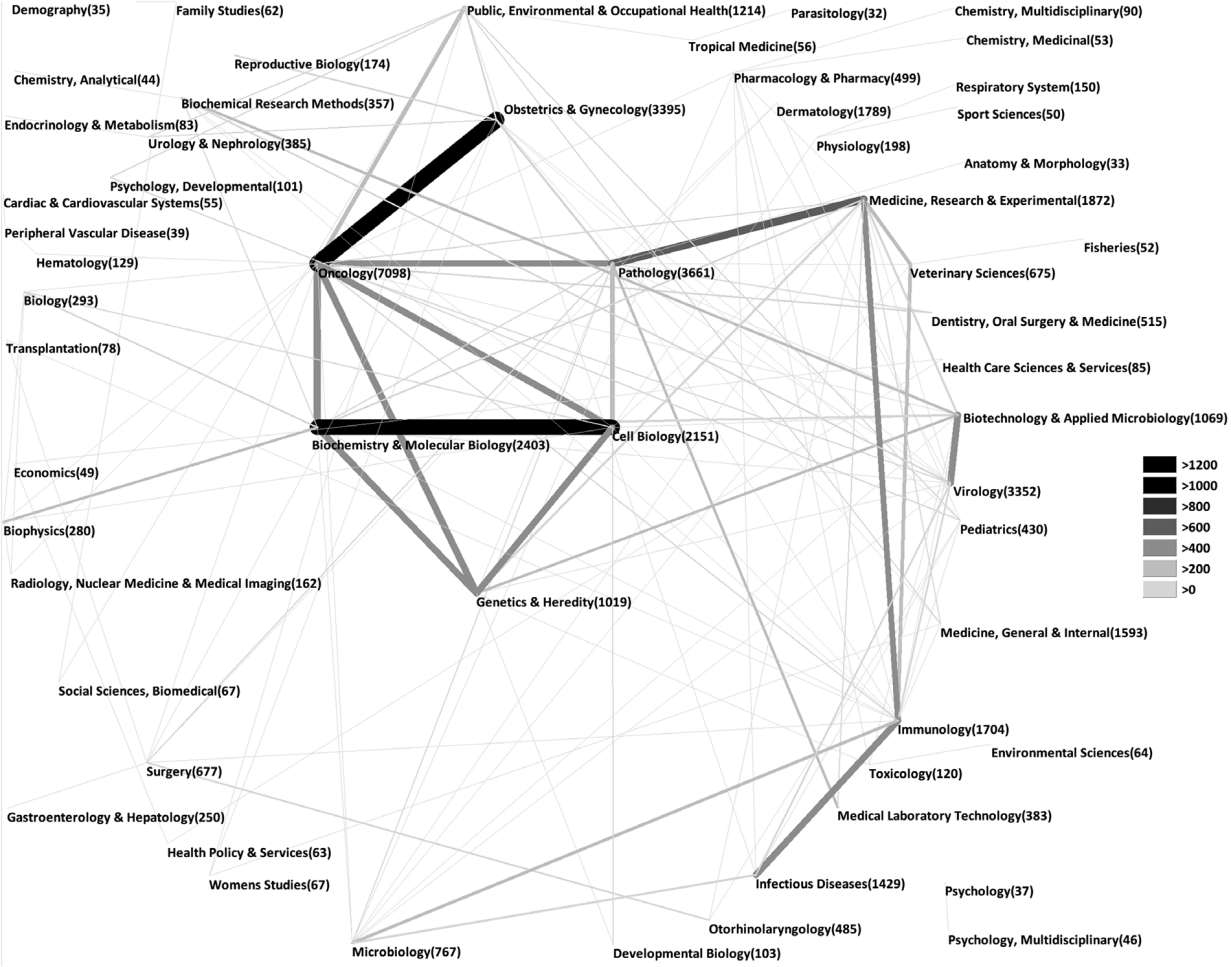
Combinations of subject areas in the field of HPV research

The proportion of the ten most frequent subject areas was also analyzed in a country-specific manner. Again, the most frequent subject category in all examined countries was “Oncology”. The category “Dermatology” was more frequently found in Germany, France and Japan in comparison to other countries. By contrast, the category “Obstetrics and Gynecology” had a high proportion in Brazil, Finland and South Korea (Figure 3). Numerous HPV-specific publications were assigned to two different subject categories indicating interdisciplinary approaches. The most common combination was “Oncology” with “Obstetrics and Gynecology” (Figure 4).

### International HPV collaborations

**Figure 5:**
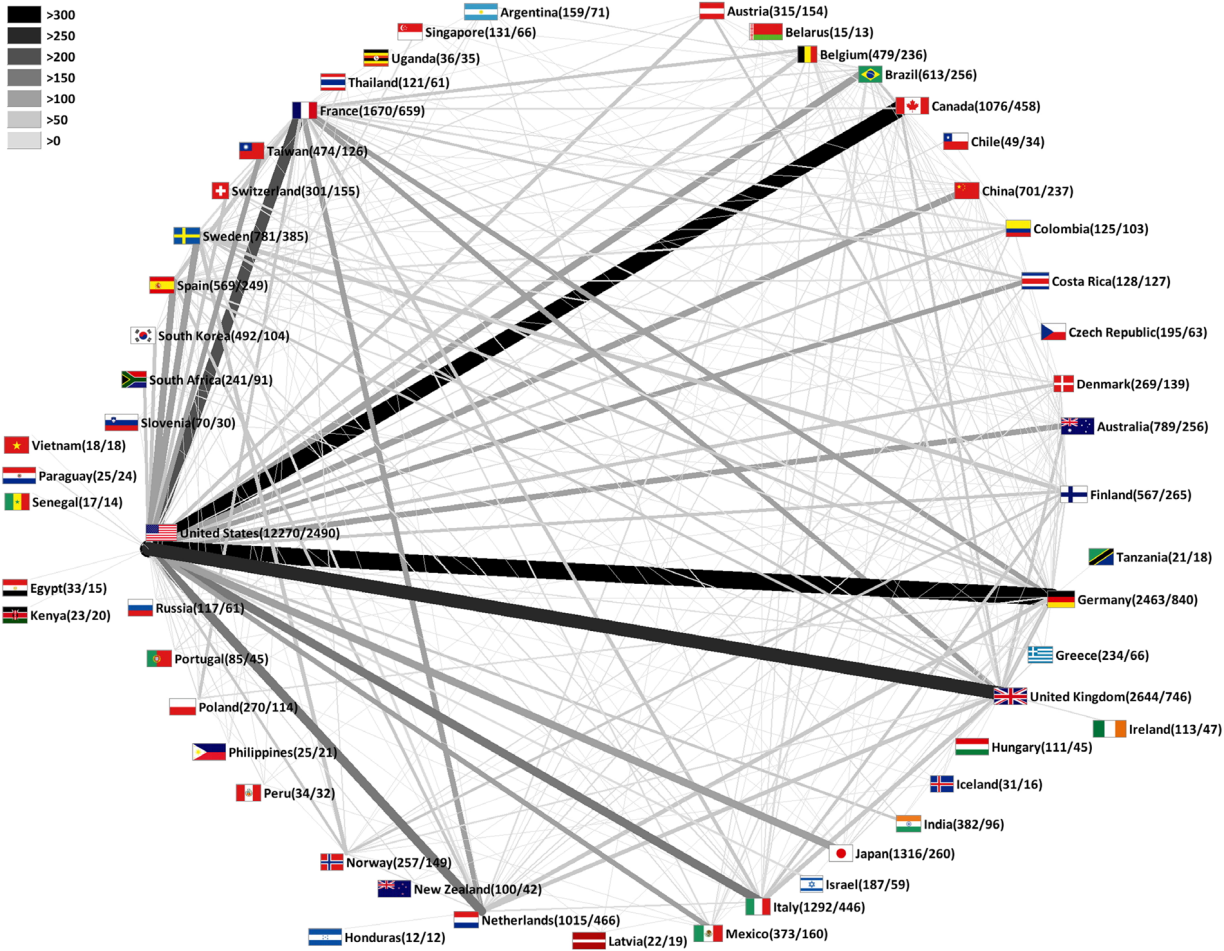
HPV research collaborations between countries Greyscale and bar thickness indicate intensity of collaborations. First ciphers in brackets indicate total publication numbers. Second ciphers indicate number of collaborative publications. Threshold: 10 collaborations of a single country.

In our investigation of countries participating in collaborative HPV research, we identified the US again in the leading position among all 158 analyzed countries. In total, US-American authors dedicated to HPV research produced 4517 collaborative items. This equates a proportion of 15.4 % of all HPV specific publications. The US was placed at the center of numerous collaborations established with nearly every other productive country. The highest level of bilateral HPV collaborations was present between the US and Germany with 332 collaborative publications (p), followed by prolific cooperations between the US and Canada (p=316), the US and the UK (p=266) as well as US and France (p=227) (Figure 5).

## DISCUSSION

The present study is the first in-depth analysis to depict the global HPV-related research architecture. It covers a period of 110 years until the Nobel prize was awarded in 2008 for the identification of HPV as a causative agent for cervical cancer. Worldwide, cervical cancer and maternal mortality each result in about 250,000 deaths per year. Also, cervical cancer causes tens of thousands of premature deaths per year in women from the most disadvantaged socioeconomic groups [12, 20]. Therefore, HPV constitutes a major global health problem. Until the development of vaccination strategies, approaches for cervical cancer control were extremely complex and expensive. Even in the current situation - with readily available HPV vaccines - cervical cancer control is a huge challenge particularly in developing nations where the disease is most prevalent [12]. To address this issue, the present study did not only cover high-income countries but focused also on middle and low-income countries. Using our validated approach, we further investigated the particular relationship between global health and economics. As hypothesized, we could detect only little research activities in most of the countries with lower economic capabilities, which served as a proxy measure for Research and Development expenditures. In this respect, cervical cancer shows a strong association with the level of development; prevalence rates were at least four-fold higher in countries ranked low in the Human Development Index (HDI) compared with those nations placed in a high category [21]. In low-resource settings, we found a considerable regional variation of cervical cancer rates with the highest numbers in Sub-Saharan Africa (24 %), Eastern Europe (21 %) and Latin America (16 %) [22, 23]. A strikingly high prevalence was also reported for Eastern Africa and the Caribbean, where rates exceed 30 % [21, 24]. For these particular regions with the greatest HPV-related burden for the female population, we could document only minimal scientific impact in the research community dedicated to HPV. This situation clearly needs to be improved. However, it does not seem realistic for these countries that basic HPV research will ever be conducted here with great success – simply due to the missing infrastructure or local personal, political or cultural challenges. But systematic public health research in the field of HPV prevention should be a realistic aim in order to rise awareness and implement standardized preventive strategies in rural and urban areas, i.e. of Sub-Saharan Africa. For instance, efforts have to be dedicated to investigate local vaccine acceptability and acceptance based on specific cultural and religious believes as a basis for effective vaccination campaigns in the future. Overall, our study reveals that most low-resource nations do not show relevant research productivity of on the quantitative level (i.e. total research activity as measured by country publications output), on the (semi-) qualitative level (i.e. measured by citation indices) and in cooperation with other. Hence, right now we simply cannot see a realistic scientific perspective for these low-income countries when the present situation remains. The gap could be bridged if non-governmental and governmental funding institutions of high-income countries direct funding into preventive strategies of HPV-caused diseases initiated in these low-income countries and also foster collaborative ties with institutions in high-income countries. By 2014, more than 57 countries included HPV vaccination strategies in their national health system programs, which is a promising step [12]. Since expansion of prevention programs to countries with the highest burden of disease is beginning [12], there is a hope that HPV related public health research will parallel this development in these countries.

Who might foster research in middle/low income countries within the area of HPV? Undoubtedly, non-governmental organizations will have to step in, e.g. the Bill and Melinda Gates Foundation. As can be seen in the latest literature, the Gates organization already focused on HPV related conditions from a global health perspective. Currently, we found 13 publications listed in the PubMed database are related to the terms “Gates foundation” AND “HPV” [25–37]. The first report in 2005 is entitled: “Coming to terms with Vietnam: the Viet/American Cervical Cancer Prevention Project” [25]. This Vietnamese-American Cervical Cancer Prevention Project had the objective to develop sustainable, cost-effective cervical cancer prevention services for women in Vietnam. The latest report estimated the value of point-of-care HPV testing in three low- and middle-income countries [37]. In its funding statement for the Alliance for Cervical Cancer Prevention, the Gates foundation awarded 50 million USD to conduct research and implement program projects in countries such as India, Mexico, Peru, Thailand, South Africa, Ecuador, Venezuela and Central America. Thereby, this funding initiative is a promising step to stimulate research in a global context that is desperately needed to address some of the shortcoming identified in our study.

In the country-specific analyses, the United States plays a dominant role within the research community dedicated to HPV. US-American authors publish the highest number of HPV-related articles, participate in the majority of international HPV collaborations, receive the highest HPV-specific h-index and obtain most citations. Among the ten most productive players regarding HPV research worldwide, only North American and Western European countries are present besides Japan and Australia. We compared the HPV-specific research activity ranking to the global scientific productivity maps describing research on other infectious agents. In one study on yellow fever disease, a total of 5,053 yellow fever-associated publications were identified, which were published by authors from 79 countries in the period of over 110 years. Similar to the present findings on HPV, the US also had the highest publication activity. However, in striking contrast to the present findings, the country with the second highest research activity was the “non-high-income” country Brazil. This is not an unique finding since Brazil also had a high research activity in leishmaniasis research as two recent studies documented. The first analysis assessed global leishmaniasis research activities between 1957 and 2006 [38]. A second study covered the interval between 1945 until 2010 and confirmed the second position of Brazilian authors among leishmaniasis researchers worldwide [39]. Apart from the dominance of the US, another exceptional picture can be drawn when tuberculosis research is evaluated: Here, US-American authors issued 11,788 items of 58,319 TBC-specific publications. The US was followed by the UK (p=4202), India (p=3456), France (p=2541), South Africa (p=1840), Germany (p=1747) and China (p=1427) [40]. When a total of 51,418 influenza virus specific publications were analyzed using a similar approach [41], the ranking of most active countries lists the US (p=19,194) at the top position, followed by the UK (p=4614), Germany (p=2951) and Japan (p=2815). In bacterial meningitis research, the US leads global research activities with a number of 2698 bacterial meningitis-related publications, followed by the UK (p=912), Germany (p=749) and France (p=620) [42].

In contrast to leishmaniasis and yellow fever, which predominantly occur in low-income countries, HPV also affects women in high-income countries. Therefore, the present data on HPV should also be compared to other infectious agents that play a major role in high-income countries. In this respect, one study focused on the Hepatitis B virus (HBV) [43] which is currently reported to be the tenth leading cause of death worldwide. In this study, scientometric methods and large-scale data analysis were used to evaluate quality and quantity of HBV research in the period between 1971 and 2011. In total 49,166 items were published by authors from 250 countries. Similar to the present study on HPV, the US was the most active country with a total of 28 % of all HBV-related publications. It was followed by Germany, China, the UK, Japan, France, Italy and Taiwan, respectively. Apart from China, no other country in the top ten was listed as an upper middle-income country, revealing similarities to the present findings.

The present socio-economic assessment of global HPV research activities indicates a prominent role of Scandinavian countries when the total publication activity is related to the GDP. Furthermore, these nations often lead the ranking when qualitative research benchmarks are evaluated (i.e. the citation rates). This finding indicates that these nations produce high quality research very effectively. Overall, middle-income countries were generally ranked lower among the power players in HPV research. This finding did not change when HPV-specific research was related to socio-economic figures, e.g. regardless of the population numbers of these countries. By contrast, when the population size-sensible index GDP/capita is used in our calculations, countries with a high population size such as China, gain importance. They occupy an outstanding position in the ranking since their GDP per capita is relatively low. However, the predominance of US-American research in the field of HPV is still undisputed. Its leading position remained unchanged when research productivity was related to socio-economic figures. For example, the US was placed in the top position when research output was seen in context to the GDP per capita as demonstrated by a value of SEI_2_=257.90. China followed with a SEI_2_ of 116.83. This finding once again underlines the highly productive US-American scientific community, which is remarkable since China lagged far behind despite its extremely low GDP per capita (China 6000 US-$ GDP/capita versus US 47,580 US-$ GDP/capita). The US-American success within the HPV research community in particular or generally in the scientific world can be attributed to the immense resources the country dedicates to Research and Development and to the well established scientific infrastructure and collaborations. For example, the Gross domestic spending on Research and Development totaled 2.74% of its GDP in 2010 (https://data.oecd.org/rd/gross-domestic-spending-on-r-d.htm). Also, US-American researchers were on the forefront of collaborative efforts not only in the field of HPV research but also in many other biomedical fields, which accelerated the US-American research productivity and the output of their partners.

## MATERIALS AND METHODS

### NewQIS protocol

To conduct this project, we used the previously described and established New Quality and Quantity Indices in Science (NewQIS) protocol aiming to analyze scientific productivity via standardized, objective and validated procedures [44, 45]. NewQIS is based upon a combination of established scientometric and economic analysis techniques; the findings were visualized via density equalizing mapping projections as employed in earlier studies [46].

### Data source

NewQIS uses the online database “Web of Science” (WoS, Thomson Scientific) [47, 48]. As an advantage, the database does not only provide data for the overall research activity analysis (publication entries) but also gives the opportunity for citation metrics in order to assess (semi)-qualitative aspects of HPV research.

### Search strategy

The search terms “HPV”, “human papilloma virus”, and “human papillomavirus” were used to identify and assess the HPV-related research activity between 1900 and 2009. We chose to evaluate this respective time period to generate an encompassing search with valid results. As observed in our other studies, the effect of the “cited half life of publications” refers to a time period of the latest years, when a decline in citations is usually seen. This finding does not represent the true recognition the publications receive in the scientific community because the time span after publication is simply too short and the items had not enough time to be cited adequately. Also, a decline of citations can be seen due to “aging” of articles. Hence, we feel the limitation of the study until 2009 was justified so the overall results will not be blurred. Also, we expect that the Nobel prize award in December 2008 will have a ripple effect on the scientific community. Therefore, the stimulated productivity needs to be analyzed to a later point in time to ensure a proper evaluation.

We used the previously mentioned search terms to conduct an all encompassing TOPIC search that identified the terms of interest in title, abstract and author key word section and identified all document types related to HPV.

### Data analysis and categorization

After retrieval of metadata of all HPV-related publications, bibliographic details were sorted and analyzed according to a number of criteria. These included the countries of origin, languages, document types, citations, cited references, year published and subject categories for all HPV-related publications. From these metadata, HPV research-specific modified country h-indices were calculated. This index was developed by Jorge Hirsch in 2005 for an assessment of the scientific quality of all items a specific author contributed to the field [49]. Here, we applied the concept to evaluate the output of single countries specifically regarding HPV research and therefore termed the parameter “modified h-index”.

### Density equalizing mapping

Density-equalizing mapping projections (DEMP) constitute a validated core technique to generate world maps that visualize geographical trends in HPV research. As described earlier [47, 50], after transfer of the bibliometric information to a data base and parameter analysis, DEMP were calculated according to the algorithm of Gastner and Newman [51], using the GeoVIZ toolkit [52]. Briefly described, the territories of the different publishing countries were separated from each other and resized in proportion to the selected criterion (i.e. country-specific h-index).

### Socio-economic analyses

In order to assess the relative contributions of the single countries towards HPV research in regard to their financial abilities, the nations´ gross domestic products (GDP) and their investments in research and development were related to their HPV research activities. Additionally, the population of the countries has been taken into account. The socio-economic numbers were obtained from the *World Bank Database* [53] and have references to the year 2009. Additionally, the categories of the World Bank have been chosen to define the different economic performances of the countries: Thus, the Gross National Income (GNI) per capita in *low-income* countries totals 1,005 USD (US-Dollar) or less. In *lower-middle-income* countries, the GNI per capita ranges from 1,006 to 3,955 USD, in *upper-middle-income* from 3,956 USD to 12,235 USD. *High-income* countries are defined by a GNI per capita of 12,236 USD and above. China’s and Taiwan’s figures has been taken from the CIA World Factbook (2008 = GDP/GDP per Capita; 2009 = Population).

### Analysis of collaborations

To analyze HPV research collaborations from a global perspective, all affiliations of the HPV-specific publications were processed as earlier described for other diseases [54, 55]. In brief, if two authors or more, who were affiliated to different countries, contributed to one HPV-specific publication, we defined this relationship as a collaboration. To visualize these interactions for single pairs of countries, vectors were generated, which were proportional in shades of grey and width to the number of established bilateral HPV collaborations [54, 55].

## CONCLUSIONS

In summary, this study is the first in depth analysis of the worldwide HPV research architecture. Using density-equalizing mapping techniques in combination with research quantity and quality indices and economic benchmarks, a global landscape of HPV research was illustrated. Our data illustrate a research activity, which largely follows the global economic situation. However, since HPV endangers wellbeing and health of women especially in low-income countries from an epidemiological perspective, these countries should be supported to participate in HPV research - predominantly in the sector of public health and prevention strategies as well as in collaborations with high-income countries.

## Abbreviations

DEMP: Density Equalizing Map Projections
GDP: Gross Domestic Product
HBV: Hepatitis B Virus
HDI: Human Development Index
HI: High-income country
HPV: Human Papilloma Virus
LMI: Lower-middle-income country
NewQIS: New Quality and Quantity Indices in Science
p: Publications
SEI_1_: Socio-Economic HPV Index 2 (Publications/GDP in 1000 bn US-$)
SEI_2_: Socio-Economic HPV Index 2 (Publications/GDP per capita in 1000 US-$)
SEI3: Socio-Economic HPV Index 3 (Publications/Population Population in mill.)
US: United States
UMI: Upper-middle-income country
WoS: Web of Science
